# Stereo Camera Head-Eye Calibration Based on Minimum Variance Approach Using Surface Normal Vectors

**DOI:** 10.3390/s18113706

**Published:** 2018-10-31

**Authors:** Joong-Jae Lee, Mun-Ho Jeong

**Affiliations:** 1 Center of Human-centered Interaction for Coexistence, 5, Hwarang-ro 14-gil, Seongbuk-gu, CHIC, Seoul 02792, Korea; arbitlee@chic.re.kr; 2 Division of Robotics, Kwangwoon University, Seoul 01890, Korea

**Keywords:** stereo camera, head-eye calibration, minimum variance approach, surface normal vector, humanoid robot

## Abstract

This paper presents a stereo camera-based head-eye calibration method that aims to find the globally optimal transformation between a robot’s head and its eye. This method is highly intuitive and simple, so it can be used in a vision system for humanoid robots without any complex procedures. To achieve this, we introduce an extended minimum variance approach for head-eye calibration using surface normal vectors instead of 3D point sets. The presented method considers both positional and orientational error variances between visual measurements and kinematic data in head-eye calibration. Experiments using both synthetic and real data show the accuracy and efficiency of the proposed method.

## 1. Introduction

Visual servoing is a fundamental skill that enables a robot to perform a variety of manipulation tasks, such as industrial automation, assistance in medical operations, and robotic grasping (i.e., service robots). It enables robot motion control by exploiting visual information extracted from a camera. Obviously, the transformation from the robot and the camera should be estimated in order to control the robot precisely. This problem, also known as hand-eye calibration, is a process that estimates the transformation between the robot end-effector and the camera mounted on it. In general, classical hand-eye calibration methods estimate the transformation between the robot end-effector and the camera coordinate systems by (1) moving the end-effector into different poses, (2) computing the transformation of the end-effector with respect to the robot base by robot kinematics, (3) detecting the calibration patterns from images, (4) calibrating the camera using a plane-based calibration method, (5) estimating the poses of the camera with respect to the calibration pattern, and (6) solving for the robot-sensor calibration.

Hand-eye calibration methods can be classified in two kinds of hardware configurations: eye-in-hand and eye-to-hand configuration. The former deals with the case where the camera is attached to a robot manipulator. The latter is for the case in which the camera is mounted on a place away from the robot. Additionally, many studies dealing with hand-eye calibration have typically been categorized in two ways: AX=XB and AX=YB problems (see details in Section 2).

**Figure 1:**
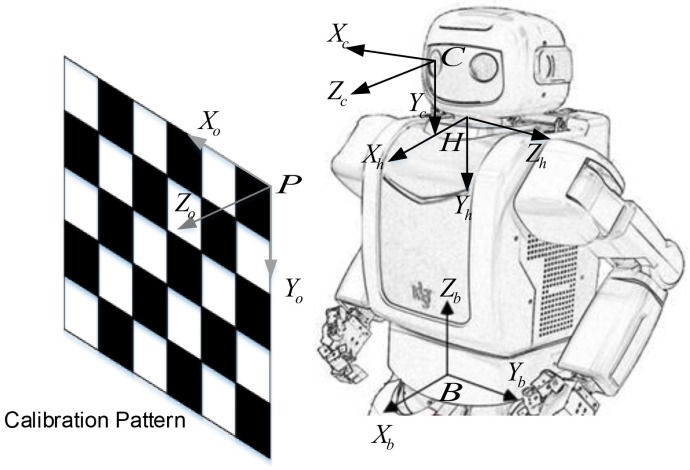
Setup for robot head-eye calibration and relevant coordinate frames: camera {*C*}, robot head (neck) {*H*}, robot base {*B*}, and calibration pattern {*P*}.

In this paper, we propose a method to determine the transformation from a robot’s head (neck) to the camera, as shown in Figure 1. It is similar to hand-eye calibration, but we call it head-eye calibration for convenience. The proposed method is an extended variance minimization (ExtMinVar) approach, which reduces the computational demand compared to the original minimum variance (MinVar) approach [1]. MinVar optimizes its solution over all 3D points in all views, which causes high amounts of computation per iteration that accumulates throughout the optimization process. The proposed ExtMinVar overcomes this issue by constructing a normal vector using 2 3D points to represent the 3D points in each view. By using only 2 3D points for each view, rather than large sets of 3D points, ExtMinVar is able to reduce the number of computations required for the coordinate transformation and covariance calculation in each iteration of the optimization, reducing the computation time while maintaining accuracy.

The main contributions of this paper are as follows:

- proposal of a cost-effective head-eye calibration that is simpler than that of the previous work [1] based on the minimum variance (MinVar) method,
- introduction of a novel cost function to minimize the covariance between surface normal vectors, rather than 3D point sets, and
- validation of the proposed approach through simulations as well as real experiments.

The rest of the paper is organized as follows: Section 2 gives an overview of the studies related to hand-eye calibration. In Section 3, we first review head-eye calibration using the MinVar approach, and our proposed method is described in detail. Section 4 shows the experimental results using both synthetic and real datasets, and the conclusion is presented in Section 5.

## 2. Related Work

Hand-eye calibration methods can be classified in several ways. One way is to distinguish whether the mathematical representations of the given problem are in the AX=XB or AX=YB form. A survey report [2] gives an overview of the methods that solve calibration problems of the forms AX=XB and AX=YB, where *X* is the unknown 4×4 hand-eye transformation, *Y* is the unknown 4×4 transformation from target to camera, and *A* and *B* are known 4×4 transformations for visual measurements and robot poses, respectively.

Another way to classify hand-eye calibration methods is by determining if they are closed-form-based or iteration-based approaches. Closed-form-based approaches use linear algebra methods to obtain the solution for hand-eye calibration. Shiu and Ahmad [3] introduced a solution with a system of rigid transformations of the form AX=XB for finding the position and rotation of a sensor with respect to the robot’s wrist. Tsai and Lenz [4] presented a similar but more efficient method for solving the hand-eye calibration problem by keeping the number of unknowns the same, regardless of the number of frames. Liang et al. [5] proposed a new method that used linear decomposition on *A* and *B* without calculating the screw vector. The closed-form based approach is simple and easy to implement, but it often does not give results that are sufficiently accurate due to noises from various sources during measurement.

On the other hand, many studies have been proposed to reduce calibration errors with iterative cost-minimization. Iteration-based approaches are robust to noise and calibration errors at all stages from sensing to calibration. Although the initial estimate is not optimal, nonlinear optimization can be applied to obtain an optimal solution by iteratively minimizing a cost function. Horaud and Dornaika [6] presented a different formulation of hand-eye calibration using 3×4 perspective matrices as well as the classical AX=XB. They also showed that the non-linear optimization method yielded more accurate results than the closed-form solution. Shi et al. [7] proposed an iterative approach that simultaneously solved both hand-eye and base-world calibrations using the quaternion representation of the rotations rather than the rotation matrix. Zhuang et al. [8] introduced a one-stage iterative method with the Levenberg–Marquardt algorithm, eliminating the propagation of rotation errors to position errors. Wei et al. [9] proposed a method of calibrating robotic hand cameras by active motion, which does not require metric control points nor the initial values of the unknowns in non-linear equations. Strobel and Hirzinger [10] presented a novel metric on the group of rigid transformation (SE(3)) to optimally estimate the parameters of the system model. It automatically adjusted the weights of the translational and rotational errors to perform an optimal estimation. In [7], an online hand-eye calibration method using motion selection was proposed, which not only prevented the degenerate cases in the calibration process, but also tried to increase calibration accuracy by choosing appropriate motions. Fassi and Legnai [11] presented the equation AX=XB of hand-to-sensor calibration as a geometrical interpretation. The form of $AX_{i}=X_{i}B$ is of an overconstrained system, and the optimal approximation can be found with the least square method, which minimizes the errors. While most methods apply L2 optimization for hand-eye calibration, Zhao [12] leveraged a convex optimization without a starting value. Hartley et al. [13] showed that conjugate rotation averaging is related to hand-eye calibration, which computes the relationship between the robot and camera frames. Heller et al. [14] considered hand-eye calibration as the minimization of the ϵ-epipolar constraint and deployed the branch-and-bound (BnB) approach to minimize the objective function. They showed that their solution is guaranteed to be globally optimal with respect to the $L_{\infty }$-norm, which is based on the BnB search over the space of rotations in [15]. Li et al. [16] presented a probabilistic approach to find the hand-eye and robot-world transformation (AX=YB problem) even when noisy scrambled data are used as input due to the asynchrony of sensor streams.

At its core, hand-eye calibration works similarly to ICP (Iterative Closest Point), where two sets of 6 DoF (positional and orientational) data are matched via 2D–2D, 2D–3D, or 3D–3D registration, depending on the dimensions of the input data. Ishikawa et al. [17] presented a 2D–3D calibration between a LiDAR and a camera, based on hand-eye calibration. The LiDAR motions are estimated by an ICP algorithm and camera motions are computed by feature point matching. Limoyo et al. [18] proposed the self-calibration of a mobile manipulator by aligning two point clouds from a depth camera and a contact sensor using an ICP-based algorithm.

This work achieves head-eye calibration for settings that can be expressed in the AX=XB form using an iteration-based optimization. We propose a simpler and more efficient computation of the original MinVar algorithm, while also maintaining its robustness to input noise and accuracy.

## 3. Overview of Robot Head-Eye Calibration

### 3.1. Problem Statement and Formulation of Head-Eye Calibration

The goal of head-eye calibration is to estimate the transformation from the robot’s head (neck) to the camera as given below:

(1) $$T_{C_{r}H}=\left(\begin{array}{cc} R_{C_{r}H} & t_{C_{r}H}\\ 0 & 1 \end{array}\right),$$

where $R_{C_{r}H}\in SO\left(3\right)$ and $t_{C_{r}H}\in R^{3}$ are the rotation and translation from the right camera to the robot’s head, respectively.

**Figure 2:**
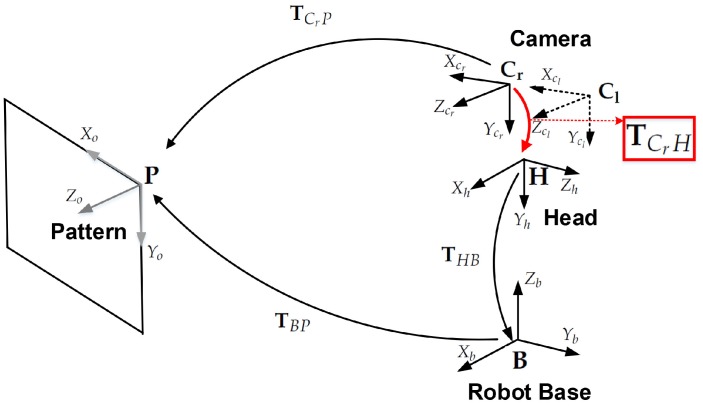
Transformations between coordinates. $T_{C_{r}H}$ is estimated in the calibration process (denoted by the red rectangle).

Figure 2 shows the transformation between coordinate systems for head-eye calibration, where each transformation is denoted as follows:

- $T_{C_{r}P}$: The transformation from the right camera to the pattern coordinate system,
- $T_{C_{r}H}$: The unknown transformation from the right camera to the robot’s head coordinate system, which will be estimated by this calibration method,
- ${\hat{T}}_{C_{r}H}$: An initial estimate of the $T_{C_{r}H}$ transformation,
- $T_{HB}$: The transformation from the robot’s head to the robot base coordinate system,
- $T_{BP}$: The transformation from the robot base to the pattern coordinate system.

To solve for head-eye calibration, three or more measurements obtained from different head poses using forward kinematics are needed. The pattern is observed from each of the different poses of the camera.

Let us suppose that the robot’s head has been turned through *n* poses with the camera measuring *m* feature locations on the pattern plane. Given the triangulated 3D points $p_{B}^{ij}$ from the stereo camera and robot head rotation angles $\left({R_{X_{C_{r}}}}^{ij},{R_{Y_{C_{r}}}}^{ij}\right)$ for every pose i=1,⋯,n and feature point j=1,⋯,m, the unknown $T_{C_{r}H}$ can be computed from an initial estimate ${\hat{T}}_{C_{r}H}$ with iterative optimization techniques. Here, we can also determine the transformation from the robot’s head to the robot base coordinate system $T_{HB}$ through robot kinematics.

### 3.2. Robot Head-Eye Calibration Based on the MinVar Approach

**Figure 3:**
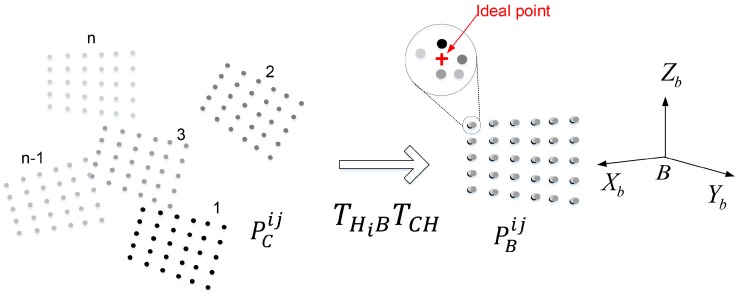
MinVar method: Transformation of each 3D point from the camera coordinate system to the robot base coordinate system.

We briefly review the MinVar method [1] for head-eye calibration. The basic idea of the MinVar method is illustrated in Figure 3, which enforces the constraint that the corresponding 3D points observed from *n* different views of a planar pattern must be mapped to the same location when transformed into the reference coordinate system. This makes it possible to find the optimal solution even in the presence of noisy data by minimizing the covariance of the 3D points.

As mentioned in Section 2, most previous works used transformations between robot end-effector motions, transformations between camera motions, and the relationship between them to find the rigid transformation of the robot’s head to the camera. There may be several numerical errors in computing the transformation, such as rounding error during matrix inversion, error in pixel localization, and quantization error on each iteration. In order to reduce these errors, the MinVar method uses the 3D point sets as inputs, rather than transformations. This allows us to decrease the number of variables to be estimated from 6×n to *n* variables when observed in *n* different views.

By moving the robot’s head in different directions, we obtain 3D points using *m* feature locations corresponding to left and right images of the stereo camera, as shown in Figure 3. Because each corresponding 3D point is obtained from different camera coordinate systems, all 3D points must be transformed to the reference coordinate system by Equation (2). The positional error can then be computed over *n* corresponding 3D points for each feature point.

(2) $$p_{B}^{ij}=T_{H_{i}B}\;T_{CH}\;p_{C}^{ij}\;\left(i=1,\ldots ,n,\;j=1,\ldots ,m\right),$$

where $p_{B}^{ij}$ denotes the 3D position of the *j*-th point at the *i*-th robot pose with respect to the robot base coordinate system, and $p_{C}^{ij}$ denotes the 3D position of the *j*-th point at the *i*-th robot pose with respect to the camera coordinate system.

However, the 3D points transformed to the robot base coordinate system may not be coincident with each corresponding point due to measurement noise. The MinVar method of finding the solution is to minimize the covariance of positional errors between the corresponding 3D points:

(3) $${\left\{T_{CH}\right\}}^{★}=\arg\min_{T_{CH}}\left(\sum_{j=1}^{m}\sum_{k=1}^{3}\lambda_{p_{B}^{j}}^{k}\right),$$

where $\lambda_{p_{B}^{j}}^{k}$ is the *k*th eigenvalue of the covariance matrix of the *j*th point set. The solution of $T_{CH}$ can be obtained by minimizing the sum of the eigenvalues of $\Sigma_{p_{B}^{j}}$.

## 4. Extended MinVar Approach Using Surface Normal Vectors

**Figure 4:**
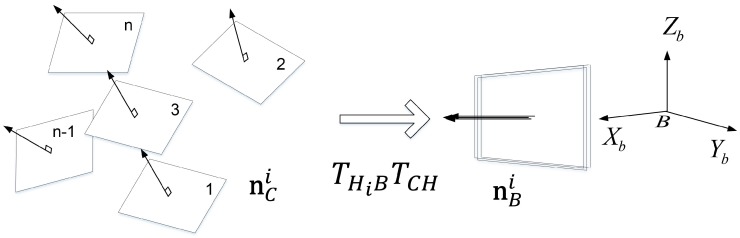
Proposed method: Transformation of each surface normal vector from camera coordinate system to robot base coordinate system.

In this section, we describe an extended version of the MinVar approach for head-eye calibration that uses surface normal vectors as its input data instead of a 3D point set, as shown in Figure 4. The computational cost of the proposed method can be decreased by avoiding the transformation of the 3D point set at every optimization step and utilizing surface normal vectors instead, which are calculated from the start.

The calibration board is geometrically represented by a 3D plane so that a set of normal vectors in the 3D plane at *n* different orientations is ideally coincident with one normal vector when transforming to the robot base coordinate system. However, error between the estimated vectors may occur in practice due to noisy input data. We estimate the variance of the error, and obtain the optimal solution by minimizing it. Using the normal vector of a 3D plane is a more simple and efficient way to find the solution than using the 3D points of the 3D plane, since it requires less coordinate transformations.

**Figure 5:**
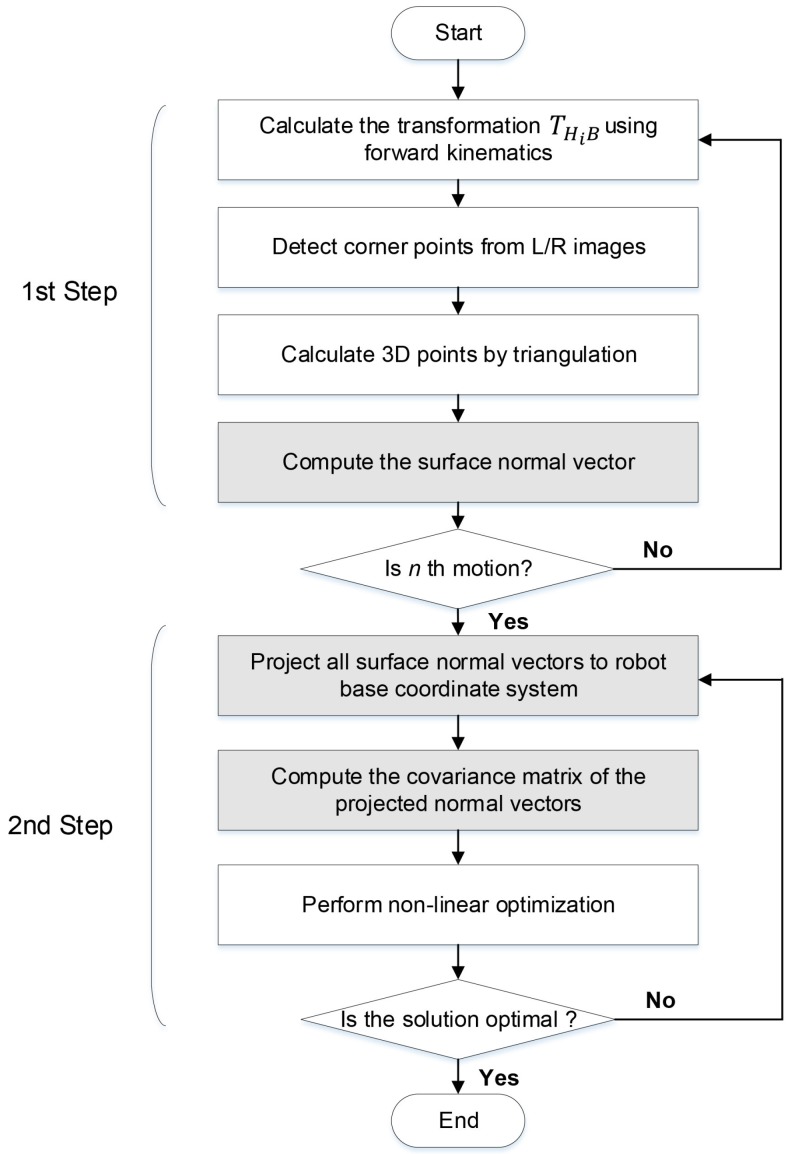
Flowchart of the proposed method. The grayed blocks denote the new steps in the proposed method compared to the previous method.

The flow chart of the proposed method consisting of the following two steps is shown in Figure 5. In the first step, we estimate transformations ($T_{H_{j}B}$) from the robot’s head to the robot base coordinate system using forward kinematics, and the surface normal vectors ($n_{C}^{j}$) of the calibration plane from stereo images according to *n* robot poses. In the second step, the estimated surface normal vectors are projected to the robot base coordinate system, and then the solution is determined by iteratively minimizing the covariance of differences between the normal vectors. We describe the procedure of our proposed method in the following sections in more detail.

### 4.1. Calculate a Transformation from Robot to Head (Neck) Using Forward Kinematics

Our head-eye calibration uses the motion of the robot’s head for kinematic measurements, such as the yaw and pitch angles of the robot’s head taken from each joint encoder. The robot head is regularly rotated in planned directions to autonomously extract kinematic measurements as well as visual measurements from multiple viewpoints.

### 4.2. Corner Detection and Triangulation

In this paper, we use a stereo camera mounted on the robot’s head rather than a monocular camera that is usually used for hand-eye calibration. By collecting 2D corner points from the images, the poses with respect to the pattern coordinate of the camera can be estimated by [19]. In contrast to this approach, a set of 3D points is exploited rather than 2D corner points in the calibration process. In fact, for visual measurements, we use surface normal vectors, which are calculated from the reconstructed 3D points through triangulation using the 2D feature points of the left and right images taken by the stereo camera. The stereo camera is assumed to be both internally and externally calibrated in this work.

We use a robust method [20] to automatically detect calibration markers in the chessboard pattern. This method involves the use of two symmetric properties of the chessboard pattern relevant to geometric and brightness distribution, and two concentric circles as a probe. In particular, it is comparatively robust to changes in pose, scale, and illumination.

**Figure 6:**
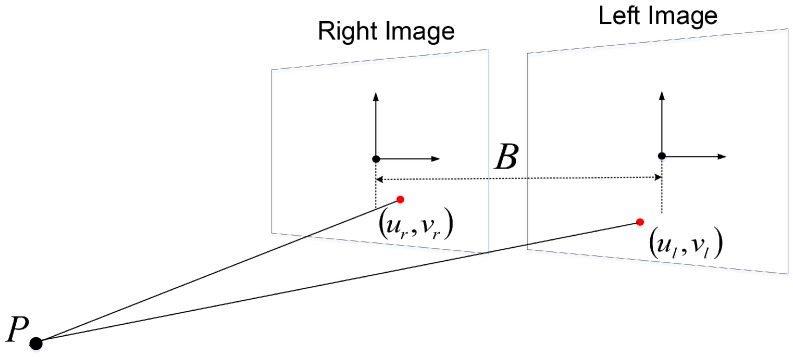
3D point reconstruction by triangulation.

As shown in Figure 6, a 3D point *P* can be calculated in homogeneous coordinates by triangulation using two corresponding 2D corner points $\left(u_{l},v_{l}\right)$ and $\left(u_{r},v_{r}\right)$:

(4) $$\begin{array}{c} P=\left(x,y,z\right)=\left(u_{l}\times \frac{z}{f},\;v_{l}\times \frac{z}{f},\;\frac{f\times B}{\left(u_{l}-u_{r}\right)},\;1\right), \end{array}$$

where *B* is the baseline between the left and right cameras and *f* is the camera focal length.

### 4.3. Surface Normal Vectors in a Camera Coordinate System

As mentioned earlier, we estimate the surface normal vector of each 3D plane, geometrically representing the calibration board, and use this feature for head-eye calibration. For each viewpoint, the surface normal vector is estimated from the 3D positions of 2D corner points on the calibration image using principal component analysis (PCA) [21]. The principal components can be obtained via singular value decomposition (SVD). In practical implementation, random sample consensus (RANSAC) is also employed to improve the robustness to noise of conventional PCA.

**Figure 7:**
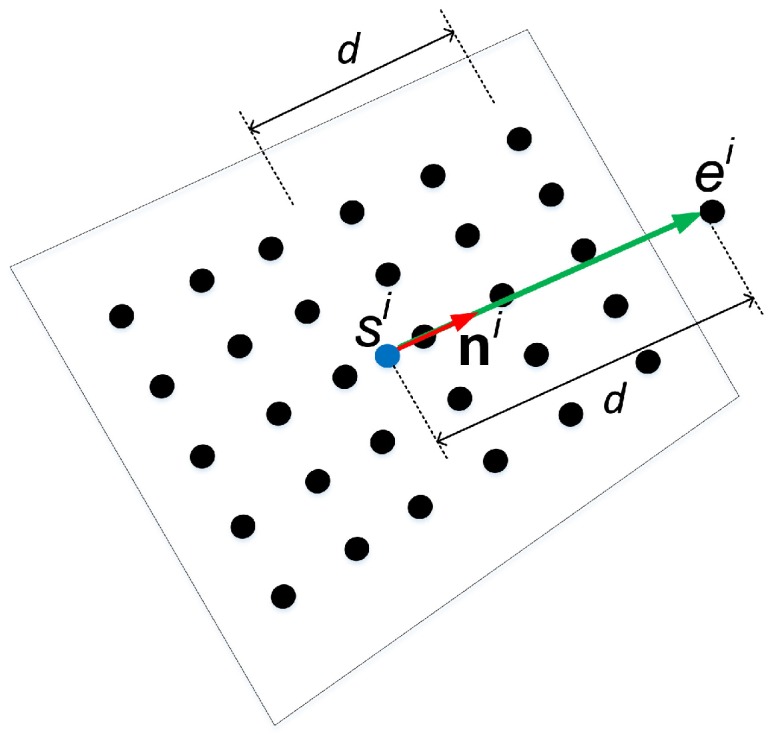
Directed line segment representation of normal vector used in this work.

The construction of normal vectors used in this work is shown in Figure 7. The surface normal we use is a directed line segment in 3D space defined by two distinct 3D points. The starting point, $s^{i}$, is located at the center of the calibration pattern plane and the ending point, $e^{i}$, is located normal to and *d* distance above the pattern plane (i.e., $e^{i}=dn^{i}+s^{i}$, where $n^{i}$ is the unit vector normal to the pattern plane). The distance *d* is set to be half the length of the longer distance between the corner feature points. This distance is defined to give weight to the actual metric of the measurement. The directed line segment is constructed by connecting from $s^{i}$ to $e^{i}$, and is the normal vector used as input for the following steps of the algorithm.

### 4.4. Transform Normal Vectors from Camera to Robot Coordinate System

After calculating the normal vector of each plane in the camera coordinate system, the set of all starting points $S_{C_{r}}$ and ending points $E_{C_{r}}$ in the camera coordinate system are transformed into the robot base coordinate system by

(5) $$\begin{array}{cc} S_{B}\left(\Theta_{C_{r}H}\right) & =\left\{s_{B,\Theta_{C_{r}H}}^{i}\;|\;s_{B,\Theta_{C_{r}H}}^{i}=T_{H_{i}B}\;T\left(\Theta_{C_{r}H}\right)\;s_{C_{r}}^{i},\;s_{C_{r}}^{i}\in S_{C_{r}}\right\},\\ E_{B}\left(\Theta_{C_{r}H}\right) & =\left\{e_{B,\Theta_{C_{r}H}}^{i}\;|\;e_{B,\Theta_{C_{r}H}}^{i}=T_{H_{i}B}\;T\left(\Theta_{C_{r}H}\right)\;e_{C_{r}}^{i},\;e_{C_{r}}^{i}\in E_{C_{r}}\right\},\;\left(i=1,\ldots ,n\right),\\ \Theta_{C_{r}H} & =\left\{\theta_{x},\theta_{y},\theta_{z},t_{C_{r}H}\left(x\right),t_{C_{r}H}\left(y\right),t_{C_{r}H}\left(z\right)\right\}, \end{array}$$

where $s_{B,\Theta_{C_{r}H}}^{i}$ and $s_{C_{r}}^{i}$ are the *i*-th starting points with respect to the robot base and camera coordinate systems, respectively, and $e_{B,\Theta_{C_{r}H}}^{i}$ and $e_{C_{r}}^{i}$ are the *i*-th ending points with respect to the robot base and camera coordinate systems, respectively. $S_{B}\left(\Theta_{C_{r}H}\right)$ and $E_{B}\left(\Theta_{C_{r}H}\right)$ are the sets of all starting and ending points in the robot base coordinates, respectively, parametrized by the transformation given by $\Theta_{C_{r}H}$, which is the vectorized 6-DOF constituents of $T_{C_{r}H}$. T(·) is the conversion function that takes a 6-DOF vector form of a transformation and converts it into a 4×4 transformation matrix as follows:

(6) $$T\left(\Theta_{C_{r}H}\right)=T_{C_{r}H}=\left[\begin{array}{cccc} & & & t_{C_{r}H}\left(x\right)\\ & R_{C_{r}H}\left(\theta_{x},\theta_{y},\theta_{y}\right) & & t_{C_{r}H}\left(y\right)\\ & & & t_{C_{r}H}\left(z\right)\\ 0 & 0 & 0 & 1 \end{array}\right].$$

This allows all normal vectors to be coincident with each other by transforming the normal vector of each plane associated with different views to the same coordinate system.

By transforming only one normal vector per view using the proposed method as opposed to transforming all 3D points in the view, ExtMinVar significantly reduces the computational cost compared to MinVar as the number of iterations for the 2nd step of the algorithm increases. Since ExtMinVar only uses a normal vector representation, only two 3D points must be transformed to the robot base coordinate system per view, as opposed to the *m* 3D points needed to be transformed per view for the MinVar approach. This also means that the covariance matrix computation and non-linear optimization in Section 4.5 only have to run over two 3D points rather than *m* 3D points per view, drastically reducing the amount of computation needed. If the initial estimate includes large errors, more iterations in the second step of this algorithm is required, so the computation time for ExtMinVar is much smaller than that of MinVar as the number of iterations increase asymptotically. The difference in required time, shown in the results in Section 5, verifies this experimentally.

### 4.5. Optimization Based on Normal Vector Variance Minimization

Ideally, all normal vectors transformed to the robot base coordinate system are necessarily coincident with each other (i.e., the sizes of $S_{B}\left(\Theta_{C_{r}H}\right)$ and $E_{B}\left(\Theta_{C_{r}H}\right)$ are each 1). Error between ideal and estimated normal vectors nevertheless occurs, due to measurement noise or various errors in the estimation process. The optimal solution is then found by a constrained minimization of this error with respect to the condition that all normal vectors are coincident with the ideal one. In general, the error between surface normal vectors is calculated as the Euclidean distance between vectors. This distance is uniformly weighted for all normal vectors, which does not accurately reflect the characteristics of the data distribution. Each normal vector must therefore be weighted by a covariance matrix that is calculated from the distribution of normal vectors about the *x*-, *y*-, and *z*-axis. The covariance matrix of all starting points, $S_{B}\left(\Theta_{C_{r}H}\right)$, is given by

(7) $$\begin{array}{c} \mathrm{Cov}\left(S_{B}\left(\Theta_{C_{r}H}\right)\right)=\left[\begin{array}{ccc} \mathrm{cov}\left(S_{B_{xx}}\left(\Theta_{C_{r}H}\right)\right) & \mathrm{cov}\left(S_{B_{xy}}\left(\Theta_{C_{r}H}\right)\right) & \mathrm{cov}\left(S_{B_{xz}}\left(\Theta_{C_{r}H}\right)\right)\\ \mathrm{cov}\left(S_{B_{yx}}\left(\Theta_{C_{r}H}\right)\right) & \mathrm{cov}\left(S_{B_{yy}}\left(\Theta_{C_{r}H}\right)\right) & \mathrm{cov}\left(S_{B_{yz}}\left(\Theta_{C_{r}H}\right)\right)\\ \mathrm{cov}\left(S_{B_{zx}}\left(\Theta_{C_{r}H}\right)\right) & \mathrm{cov}\left(S_{B_{zy}}\left(\Theta_{C_{r}H}\right)\right) & \mathrm{cov}\left(S_{B_{zz}}\left(\Theta_{C_{r}H}\right)\right) \end{array}\right], \end{array}$$

where $\mathrm{Cov}(S_{B}\left(\Theta_{C_{r}H}\right))$ is the covariance matrix of all starting points with respect to the robot base coordinate system at the *k*th iteration and $\mathrm{cov}\left(X\right)$ denotes the covariance of *X*. This is also done to the ending points to calculate the covariance matrix $\mathrm{Cov}(E_{B}\left(\Theta_{C_{r}H}\right))$ of all ending points $E_{B}\left(\Theta_{C_{r}H}\right)$ with respect to the robot base coordinate system. In Equation (7), each entry of the covariance matrix is calculated as:

(8) $$\begin{array}{c} \left\{\begin{array}{c} \mathrm{cov}\left(S_{B_{xx}}\right)=E\left[\left(s_{B_{x}}^{i}-\bar{s_{B_{x}}}\right)\left(s_{B_{x}}^{i}-\bar{s_{B_{x}}}\right)\right]\\ \mathrm{cov}\left(S_{B_{xy}}\right)=E\left[\left(s_{B_{x}}^{i}-\bar{s_{B_{x}}}\right)\left(s_{B_{y}}^{i}-\bar{s_{B_{y}}}\right)\right]\\ \mathrm{cov}\left(S_{B_{xz}}\right)=E\left[\left(s_{B_{x}}^{i}-\bar{s_{B_{x}}}\right)\left(s_{B_{z}}^{i}-\bar{s_{B_{z}}}\right)\right]\\ \mathrm{cov}\left(S_{B_{yx}}\right)=E\left[\left(s_{B_{y}}^{i}-\bar{s_{B_{y}}}\right)\left(s_{B_{x}}^{i}-\bar{s_{B_{x}}}\right)\right]\\ \mathrm{cov}\left(S_{B_{yy}}\right)=E\left[\left(s_{B_{y}}^{i}-\bar{s_{B_{y}}}\right)\left(s_{B_{y}}^{i}-\bar{s_{B_{y}}}\right)\right]\;\left(i=1,\ldots ,n\right),\\ \mathrm{cov}\left(S_{B_{yz}}\right)=E\left[\left(s_{B_{y}}^{i}-\bar{s_{B_{y}}}\right)\left(s_{B_{z}}^{i}-\bar{s_{B_{z}}}\right)\right]\\ \mathrm{cov}\left(S_{B_{zx}}\right)=E\left[\left(s_{B_{z}}^{i}-\bar{s_{B_{z}}}\right)\left(s_{B_{x}}^{i}-\bar{s_{B_{x}}}\right)\right]\\ \mathrm{cov}\left(S_{B_{zy}}\right)=E\left[\left(s_{B_{z}}^{i}-\bar{s_{B_{z}}}\right)\left(s_{B_{y}}^{i}-\bar{s_{B_{y}}}\right)\right]\\ \mathrm{cov}\left(S_{B_{zz}}\right)=E\left[\left(s_{B_{z}}^{i}-\bar{s_{B_{z}}}\right)\left(s_{B_{z}}^{i}-\bar{s_{B_{z}}}\right)\right] \end{array}\right. \end{array}$$

where E(·) denotes the expected mean of its argument. In addition, $s_{B_{x}}^{i}$, $s_{B_{y}}^{i}$, $s_{B_{z}}^{i}$ are respectively the *x*-, *y*-, *z*- components of the *i*th plane center and $\bar{s_{B_{x}}}$, $\bar{s_{B_{y}}}$, $\bar{s_{B_{z}}}$ are respectively the mean of the *x*, *y*, and *z* components of the *i*th plane center. The same formula is used for the ending points with the corresponding changes in input variables.

Normally, the initial estimate ${\hat{T}}_{C_{r}H}$ of $T_{C_{r}H}$ is not optimal, and thus we must compute the optimal solution through a non-linear optimization that minimizes the error between normal vectors. This requires a measure that corresponds to the uncertainty of the covariance matrix $\mathrm{Cov}(S_{B}\left(\Theta_{C_{r}H}\right))$ in Equation (7). We choose the Schatten *p*-norm of $\sqrt{\mathrm{Cov}(S_{B}\left(\Theta_{C_{r}H}\right))}$, which is known as a class of discriminative metrics of matrices [22], as an uncertainty measure. Formally, the Schatten norm for a matrix $X\in R^{m\times n}$ can be represented as:

(9) $${∥X∥}_{p}={∥\sigma \left(X\right)∥}_{p},$$

where $\sigma \left(X\right)$ is the singular value vector of matrix *X*.

Using the Schatten 2-norm metric, we define a cost function *J* that measures the averaged uncertainty of covariance matrices $\mathrm{Cov}(S_{B}\left(\Theta_{C_{r}H}\right))$ and $\mathrm{Cov}(E_{B}\left(\Theta_{C_{r}H}\right))$ of the projected normal vectors:

(10) $$\begin{array}{c} {\Theta_{C_{r}H}}^{★}=\arg\min_{\Theta_{C_{r}H}}J\left(\Theta_{C_{r}H},S_{C_{r}},E_{C_{r}}\right)=\frac{1}{2}\arg\min_{\Theta_{C_{r}H}}\left\{{∥\mathrm{Cov}\left(S_{B}\left(\Theta_{C_{r}H}\right)\right)∥}_{2}+{∥\mathrm{Cov}\left(E_{B}\left(\Theta_{C_{r}H}\right)\right)∥}_{2}\right\}, \end{array}$$

where $\mathrm{Cov}(S_{B}\left(\Theta_{C_{r}H}\right))$ and $\mathrm{Cov}(E_{B}\left(\Theta_{C_{r}H}\right))$ are the covariance matrices of the set of starting and ending points, respectively, in the robot base coordinates parametrized by $\Theta_{C_{r}H}$, and ${\Theta_{C_{r}H}}^{★}$ is the optimal solution of $\Theta_{C_{r}H}$.

After the cost function is defined as above, we can perform a nonlinear optimization to find the optimal solution ${\Theta_{C_{r}H}}^{★}$ in Equation (10). Most non-linear optimization methods can be divided into *gradient search*- and *direct search*-based optimizations. The former exploits the differentiation of the objective function to determine the direction of the optimization process. The latter is used for the case that does not require differentiation of the objective function, but uses instead the iteration memory during the optimization. For simplicity, the optimization in this paper is performed using Powell’s method [23], which is one of the direct search methods.

## 5. Experimental Results

We carried out both simulated and real experiments to validate the performance of the proposed method. To measure the error of head-eye calibration, we computed the difference ($\Delta T_{C_{r}H}={\left(T_{C_{r}H}^{ref}\right)}^{-1}T_{C_{r}H}$) between the reference transformation ($T_{C_{r}H}^{ref}$) and the estimated transformation ($T_{C_{r}H}$), as in [24]. For $\Delta T_{C_{r}H}$, the orientation error $\Delta R_{C_{r}H}$ was calculated in terms of angular error using Rodrigues’s rotation formula [25], and the translation error was measured by $\Delta t_{C_{r}H}$ in terms of absolute length.

In this experiment, we compared the closed-form and the iteration-based methods. Four closed-form methods were used, those from Park et al. [26], Liang et al. [5], Horaud et al. [6], and Chou et al. [27], whereas three iterative methods were used, which were MinVar [1], ExtMinVar by PCA, and ExtMinVar by RANSAC PCA (the latter two methods are proposed in this paper). The experiments were run on a desktop computer equipped with an i7-4790 Intel CPU with 32 GB of memory running on Windows 8.1. MATLAB was used for implementing the closed-form-based methods, and Visual Studio C++ was used for developing the iteration-based methods.

### 5.1. Simulation Results

For the simulation experiments, we generated a set of data that consisted of synthetic feature locations on images obtained from different viewpoints. The patterns on the calibration board had 40 (8×5) points, and the grid size of each pattern was 50×50 mm. The stereo camera had a spatial resolution of 320×240 pixels for both left and right cameras.

**Figure 8:**
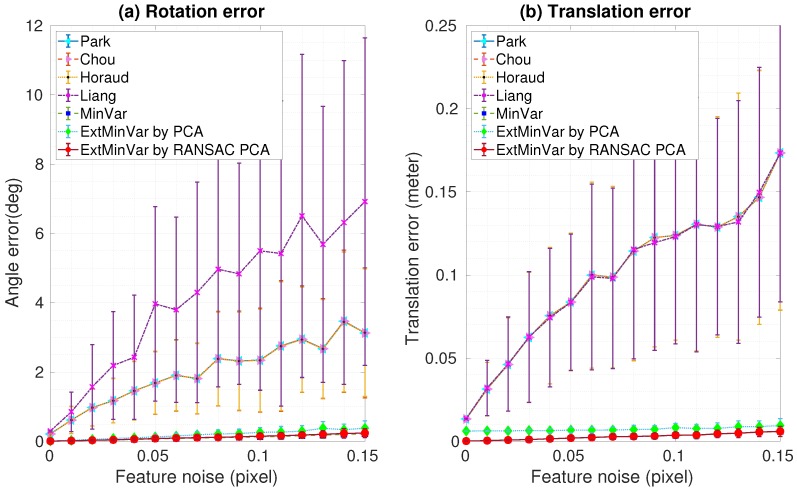
Experimental results using synthetic data with feature noise [0–0.15 pixel]: (**a**) Rotation error; (**b**) Translation error.

**Figure 9:**
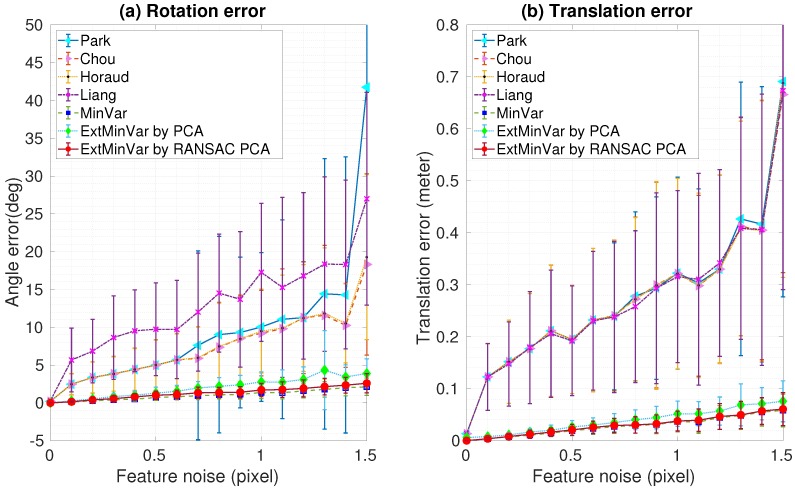
Experimental results using synthetic data with feature noise [0–1.5 pixel]: (**a**) Rotation error; (**b**) Translation error. PCA: principal component analysis; RANSAC: random sample consensus.

Robustness analysis in the presence of varying noise levels was performed by adding Gaussian noise to the ground truth data of the feature locations. Two datasets with different noise levels were generated for 100 trials in each experiment. We computed the mean and standard deviation of the rotation and translation errors, as shown in Figure 8 and Figure 9. As one might expect, both rotation and translation errors increased as the measurement noise increased. These experimental results demonstrated that the iteration-based methods showed better performances than those of the closed-form based methods. Furthermore, our proposed method showed similar results to those of MinVar, which outperformed other calibration methods. As mentioned before, PCA was used to compute the surface normal vectors in the proposed method, but it is known to be sensitive to outliers. The ExtMinVar by PCA method showed some degradation in accuracy compared to the MinVar method. However, improved accuracy could be obtained with the removal of contaminated data prior to the estimation of the normal vector. In practice, we removed outliers by applying a robust regression technique, namely RANSAC.

Figure 8 shows the rotational and translational estimation errors at varying noise levels in the first dataset. We obtained results with an error of less than 0.5 degrees of rotation, and less than 1 mm in translation.

In Figure 9, the averaged errors did not exceed a maximum of 2 degrees rotation and 5 mm translation in the second data set, though the performance was slightly degraded due to the increased noise levels.

**Figure 10:**
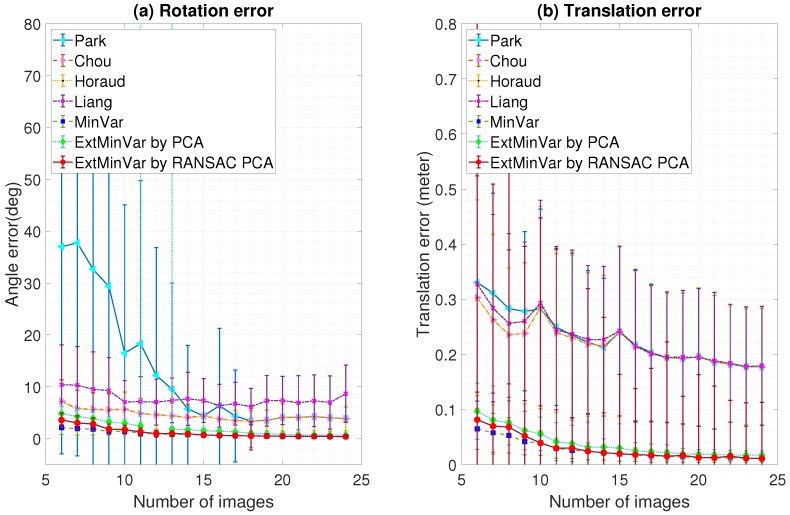
Stability of the estimated transformation with respect to the number of input images (feature noise: 0.5 pixel): (**a**) Rotation error; (**b**) Translation error.

One can expect that a more stable estimate of the head-eye calibration would be achieved as more poses are used. Figure 10 shows that the number of captured images also affected the measurement accuracy. It shows a clear trend: as more input data was provided, the more stable and accurate the results became.

**Figure 11:**
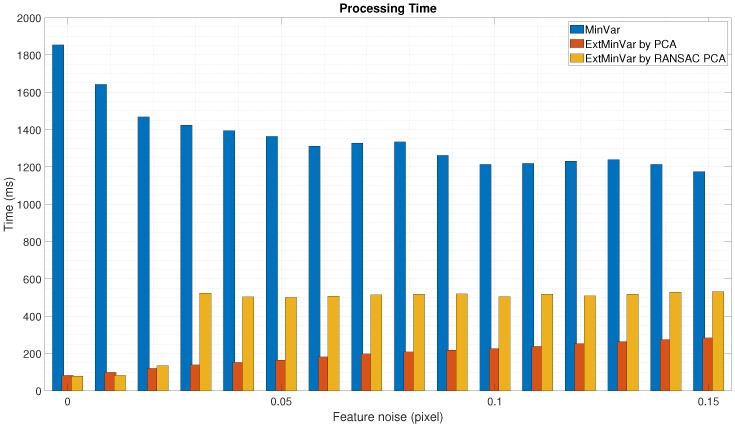
Experimental results using synthetic data with feature noise [0–0.15 pixel]: Processing time during calibration.

**Figure 12:**
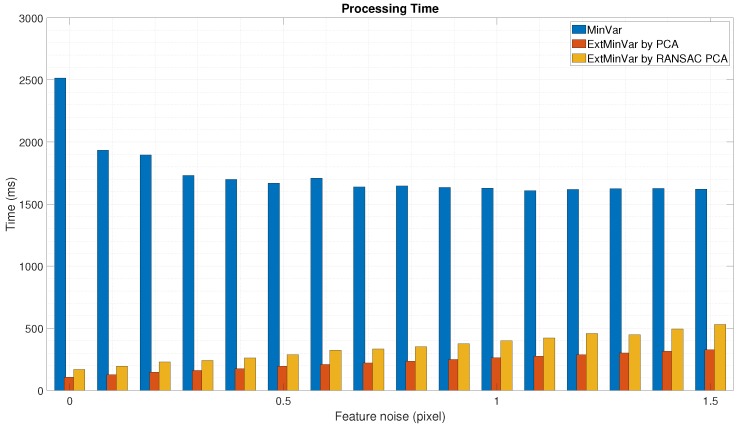
Experimental results using synthetic data with feature noise [0–1.5 pixel]: Processing time during calibration).

We also measured the elapsed time to execute the calibration procedure. It was more efficient to use surface normal vectors instead of utilizing 3D points when the calibration was performed, as shown in Figure 11 and Figure 12. In both cases, the proposed method was at least 3 to 4 times faster than the MinVar method.

**Figure 13:**
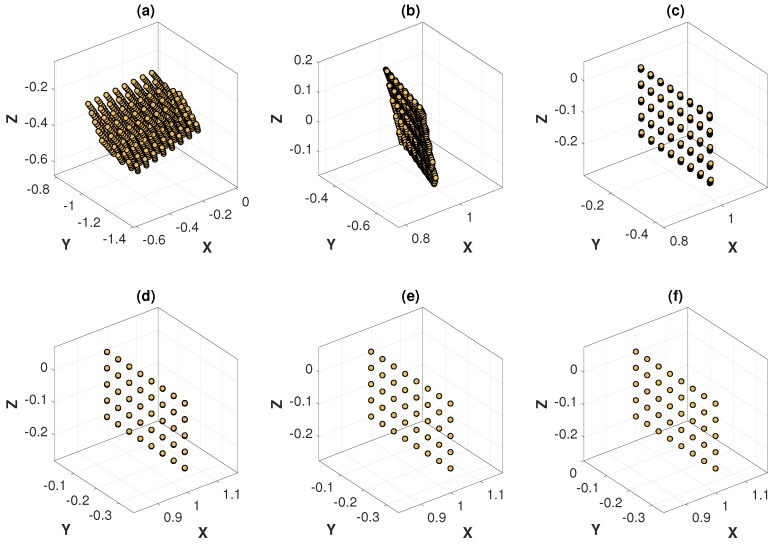
Optimization process of the MinVar method with feature noise [0–0.15 pixel]: (**a**) 3D points at iteration 1; (**b**) 3D points at iteration 6; (**c**) 3D points at iteration 10; (**d**) 3D points at iteration 13; (**e**) 3D points at iteration 16; (**f**) 3D points at iteration 27 (converged).

**Figure 14:**
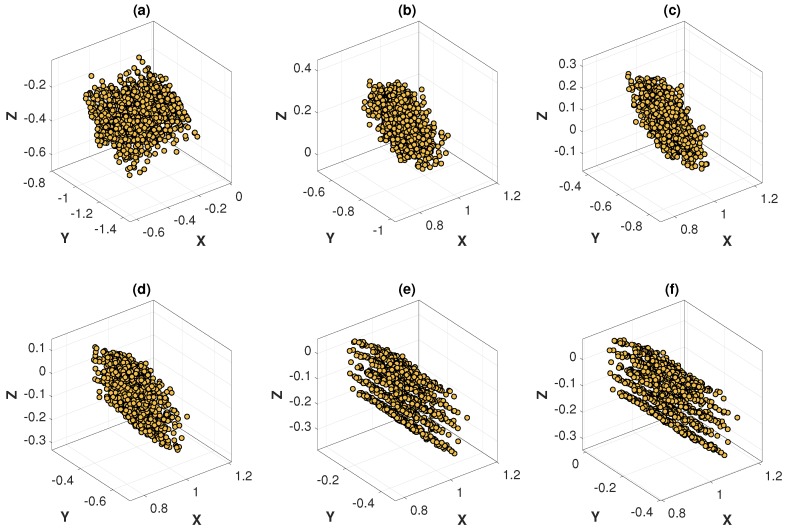
Optimization process of the MinVar method with feature noise [0–1.5 pixel]: (**a**) 3D points at iteration 1; (**b**) 3D points at iteration 3; (**c**) 3D points at iteration 5; (**d**) 3D points at iteration 7; (**e**) 3D points at iteration 10; (**f**) 3D points at iteration 15 (converged).

The optimization process of the MinVar method at different noise levels are graphically illustrated in Figure 13 and Figure 14, which are helpful for understanding how the variance of 3D points is minimized at each iteration. As expected, the variance of 3D points at convergence differed depending on the noise level.

**Figure 15:**
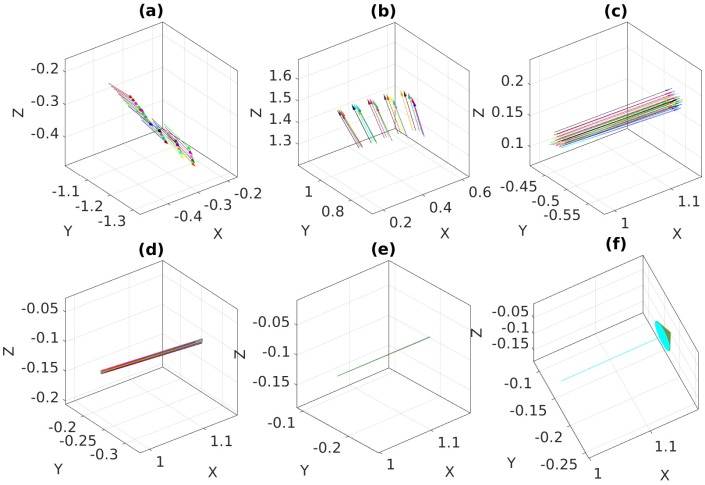
Optimization process of the proposed method with feature noise [0–0.15 pixel]: (**a**) Normal vectors at iteration 1; (**b**) Normal vectors at iteration 2; (**c**) Normal vectors at iteration 3; (**d**) Normal vectors at iteration 8; (**e**) Normal vectors at iteration 13; (**f**) Normal vectors at iteration 20 (converged).

**Figure 16:**
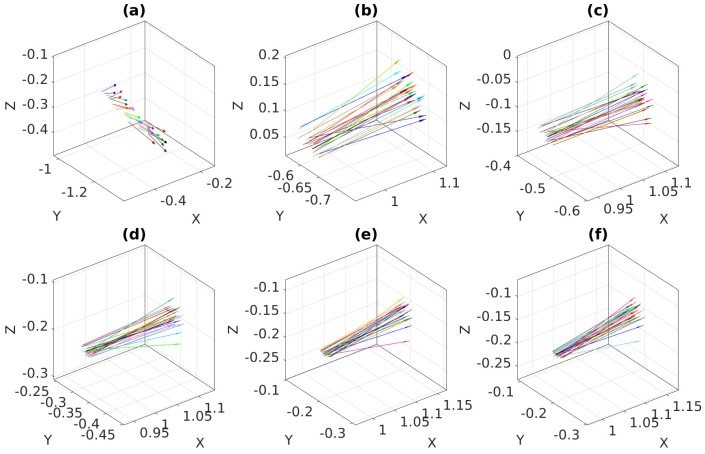
Optimization process of the proposed method with feature noise [0–1.5 pixel]: (**a**) Normal vectors at iteration 1; (**b**) Normal vectors at iteration 4; (**c**) Normal vectors at iteration 6; (**d**) Normal vectors at iteration 8; (**e**) Normal vectors at iteration 12; (**f**) Normal vectors at iteration 16 (converged).

Figure 15 and Figure 16 show the optimization process of the proposed method, which, similar to the MinVar method, minimizes the variance of normal vectors over different noise levels. After minimization, we observed that all normal vectors were coincident with each other in Figure 15f.

In contrast with the previous result, it should be noted that the normal vectors in the final iteration were not coincident due to the inherent uncertainty in the output of PCA with high levels of noise.

### 5.2. Real Experimental Results with Humanoid Robot MAHRU-Z

For the real data experiment, we used a humanoid robot named MAHRU-Z, which can perform various tasks in a home environment, as an experimental platform. In particular, MAHRU-Z provides practical domestic services based on dexterous object manipulation. It is able to articulate with 6-DOF for each arm and 2-DOF for the neck. A bumblebee stereo camera was mounted on the head of the robot for visual information (Bumblebee2, PointGrey Research, 320×240 pixels). The intrinsic parameters of the camera were fully calibrated according to [19]: the focal length was 404.409 in pixels, the camera’s principal point was (61.371, 119.874) in pixels, and the base line was 0.1199 m.

**Figure 17:**
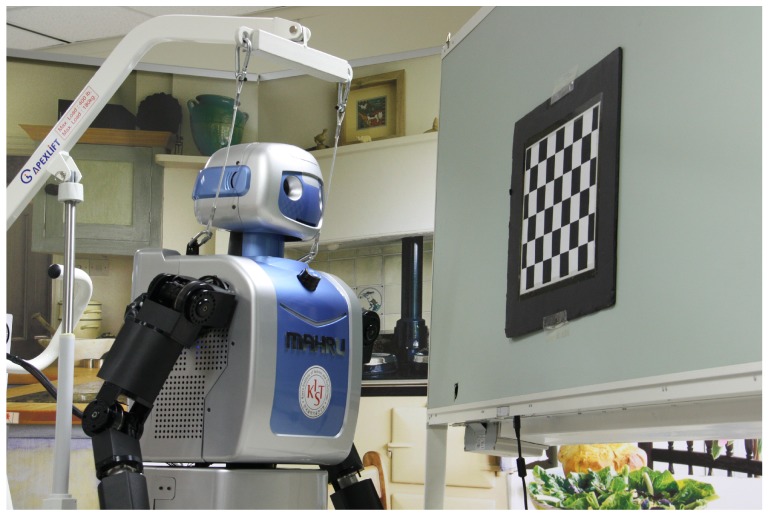
Real experimental setup: A calibration board and a stereo camera mounted on the humanoid robot, MAHRU-Z.

The reliability in grasping objects depends on an accurate calibration from the robot’s head to the stereo camera. Figure 17 shows the experimental setup for the head-eye calibration from the robot’s head to the stereo camera. We developed an automatic procedure to calibrate the relationship between the robot’s head and its eye by moving the robot’s head in 25 pre-planned directions, capturing left and right pattern images, and extracting the feature locations from them.

**Figure 18:**
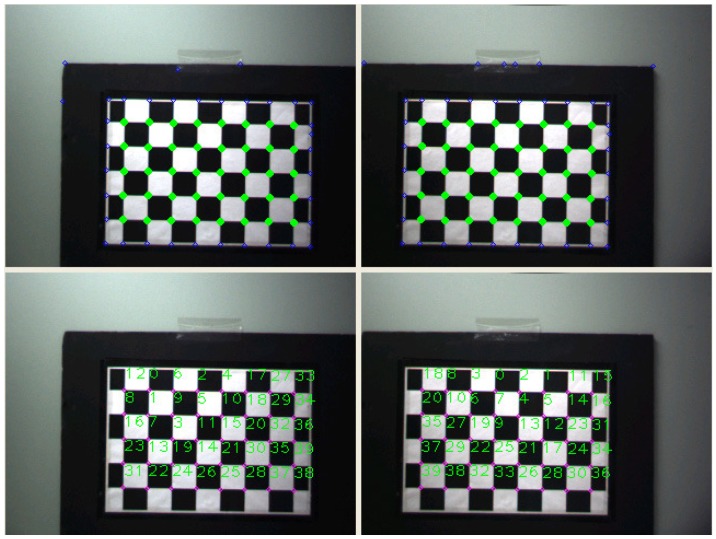
Examples of captured stereo images and their feature locations extracted through the automatic detection process.

Figure 18 shows the process of detecting feature points on the calibration pattern and finding stereo correspondences between the left and right pattern images.

Experiments were performed using the same data for five different initial estimates selected from a random interval from −20 to 20 degrees of rotation and from −60 to 30 cm of translation, and the results of the proposed method and the MinVar method were compared. The calculated transformations were all very similar, but as the initial estimation had a larger error, the processing time increased. This is due to the increase in computation time required by the optimization.

To compare the performance of the proposed method and the MinVar method, we computed the sum of variance for the 3D points transformed by each $T_{C_{r}H}$ to the robot base coordinate system and measured its processing time, shown in Table 1. We also compared the results of our proposed method against the *qhec* (quaternion hand-eye calibration) and *dqhec* (dual quaternion hand-eye calibration) optimization methods obtained from the open source implementation provided by Heller et al. [28]. However, both *qhec* and *dqhec* methods implemented in MATLAB were excluded from the comparison of processing time. Similar results were shown for the MinVar method and the ExtMinVar by RANSAC PCA compared to the *qhec* and *dqhec*. Although the variance of the ExtMinVar by PCA method was slightly larger than the others, the proposed method outperformed, in terms of processing time, those obtained by using the MinVar method.

After each calibration, we obtained the optimal transformations from the robot’s head to its eye, which were $T_{C_{r}H}^{1}$ for MinVar, $T_{C_{r}H}^{2}$ for ExtMinVar by PCA, $T_{C_{r}H}^{3}$ for ExtMinVar by RANSAC PCA, $T_{C_{r}H}^{4}$ for qhec, and $T_{C_{r}H}^{5}$ for dqhec.

$$\begin{array}{c} T_{C_{r}H}^{1}=\left[\begin{array}{cccc} -0.1442 & 0.0010 & 0.9895 & 0.0724\\ -0.0115 & 0.9999 & -0.0118 & -0.0238\\ -0.9895 & -0.01301 & -0.1441 & 0.1574\\ 0 & 0 & 0 & 1 \end{array}\right], \end{array}$$

$$\begin{array}{c} T_{C_{r}H}^{2}=\left[\begin{array}{cccc} -0.0881 & -0.1154 & 0.9894 & 0.0964\\ 0.01573 & 0.9930 & 0.1172 & -0.0596\\ -0.9960 & 0.0259 & -0.0857 & 0.0735\\ 0 & 0 & 0 & 1 \end{array}\right], \end{array}$$

$$\begin{array}{c} T_{C_{r}H}^{3}=\left[\begin{array}{cccc} -0.1256 & -0.0581 & 0.9904 & 0.0921\\ -0.0003 & 0.9983 & 0.0585 & -0.0278\\ -0.9921 & 0.0071 & -0.1254 & 0.1664\\ 0 & 0 & 0 & 1 \end{array}\right], \end{array}$$

$$\begin{array}{c} T_{C_{r}H}^{4}=\left[\begin{array}{cccc} -0.0142 & 0.0005 & 0.9999 & 0.0591\\ -0.0005 & 1.0000 & -0.0005 & -0.0522\\ -0.9999 & -0.0005 & -0.0142 & 0.0798\\ 0 & 0 & 0 & 1 \end{array}\right], \end{array}$$

$$\begin{array}{c} T_{C_{r}H}^{5}=\left[\begin{array}{cccc} -0.0147 & 0.0006 & 0.9999 & 0.0583\\ -0.0012 & 1.0000 & -0.0005 & -0.0523\\ -0.9999 & -0.0011 & -0.0147 & 0.0796\\ 0 & 0 & 0 & 1 \end{array}\right]. \end{array}$$

**Figure 19:**
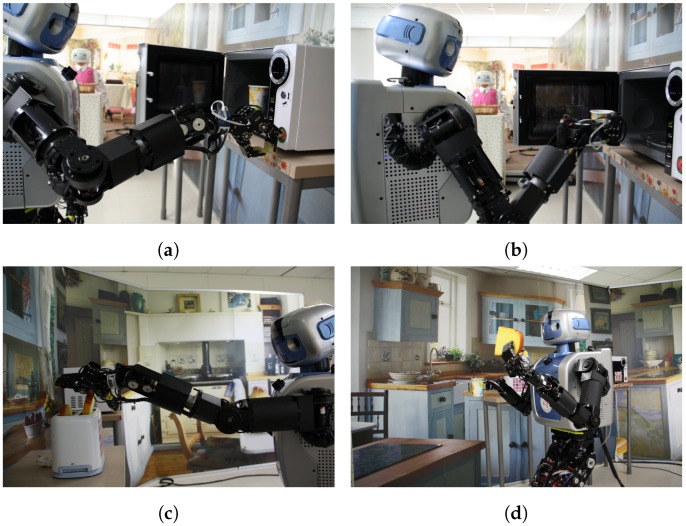
Screenshot of robot tasks in a domestic environment: (**a**) Push a button; (**b**) Grasp a cup; (**c**) Grasp toast; (**d**) Deliver the toast to a person.

Based on the results of the head-eye calibration, the robot accomplished various domestic tasks, such as pushing a button on a microwave, grasping a cup, and delivering toast, as shown in Figure 19.

## 6. Conclusions

We introduced an approach to head-eye calibration, which manages the problem of determining the geometric relationship between a robot’s head (neck) and eye (camera). This head-eye calibration is a practical example of how hand-eye calibration could be applied to real-world problems.

The proposed method is an extension of the MinVar approach, which uses surface normal vectors to reduce the large amounts of computation that using sets of 3D points brings. A central concept in the proposed method is the application of the constraint that all normal vectors transformed to the robot base coordinate system are coincident with each other. The solution was obtained by minimizing the covariance between normal vectors, and could be optimized with respect to measurement errors by imposing the constraint on all normal vectors. This method eliminates the need to transform all 3D points, triangulated on the calibration plane, to the robot base coordinate system in different viewpoints at every iteration of the optimization. This allows calibration to be performed more efficiently than in previous methods because it requires far fewer coordinate transformations, covariance calculations, and optimization searches. Furthermore, this method provides robustness to noisy measurements by minimizing the variance instead. Non-linear optimization is used to iteratively minimize the cost function and determine the transformation from the robot’s head to the stereo camera.

We showed that our proposed method provides effective and robust results on both synthetic and real data. Experimental results also showed that a humanoid robot could perform various tasks in a domestic environment when the calibration results of the proposed method were applied to visual servoing.

## Figures and Tables

**Table 1:** Results of robot head-eye calibration.

No.	$R_{0}$ (degree)	$T_{0}$ (mm)	Method (Variance (mm)/Processing time (ms))				
			MinVar	ExtMinVar by PCA	ExtMinVar by R-PCA	*qhec*	*dqhec*
1	(0, 0, 0)	(0, 0, 0)	9.35/305	13.20/22	10.0/89	9.45/-	9.45/-
2	(1, −5, −3)	(6, 30, 3)	9.25/591	13.19/24	9.81/131	8.81/-	8.79/-
3	(10, −15, 3)	(6, 11, 1)	9.27/325	9.43/34	9.73/93	8.82/-	8.79/-
4	(−10, 10, 10)	(−60, −11, −1)	8.56 /991	13.21/30	9.40/248	8.80/-	8.78/-
5	(−20, 20, −10)	(20, −10, 10)	8.65/1689	13.24/26	9.31/375	8.81/-	8.79/-
